# Influence of intraoral scanning on the quality of preparations for all-ceramic single crowns

**DOI:** 10.1007/s00784-020-03316-2

**Published:** 2020-05-20

**Authors:** Oliver Schubert, Kurt-Jürgen Erdelt, Roland Tittenhofer, Jan Hajtó, Alexander Bergmann, Jan-Frederik Güth

**Affiliations:** 1 Department of Prosthetic Dentistry, University Hospital, LMU Munich, Goethestraße 70, 80336 Munich, Germany; 2 Alpenpraxis Miesbach, Fraunhoferstr 10, 83714 Miesbach, Germany; 3 Dental Team Dr. Hajtó, Briennerstr 7, 80333 Munich, Germany

**Keywords:** CAD/CAM, Digital evaluation, All-ceramic, Preparation design, Intraoral scan (IOS)

## Abstract

**Objectives:**

To evaluate the influence of intraoral scanning on the quality of preparations for all-ceramic single crowns.

**Material and methods:**

A total of 690 randomly selected and anonymized in vivo single crown preparations were examined. Three hundred twenty-three preparations were directly recorded with an intraoral scanner (group IS). Data from plaster casts digitized by a laboratory scanner (group ID; *N* = 367) served as control. Comparisons included convergence angle, marginal design, marginal substance reduction, homogeneity of the finish line, and undercuts. Evaluation was performed using fully automated specialized software. Data were analyzed applying Kolmogorov-Smirnov, Mann-Whitney *U* test, and Fisher’s exact test. Level of significance was set at *p* < 0.05.

**Results:**

Convergence angle was above optimum in both groups, but significantly larger for group IS (*p* < 0.001). Marginal design was more ideal in group IS concerning the absence of featheredge design (*p* < 0.001) and reverse bevel (*p* = 0.211). Marginal substance reduction was closer to prerequisites for all-ceramic restorations in group IS (*p* < 0.001). Finish lines were more homogeneous in group IS regarding the uniformity of their course (*p* < 0.001). Undercuts were more frequently found in group ID than in group IS (*p* < 0.001).

**Conclusions:**

Intraoral scanning of prepared teeth has positive impact on the quality of preparations for all-ceramic single crowns regarding marginal substance reduction, marginal design, homogeneity of the finish line, and undercuts.

**Clinical relevance:**

Accurate preparation design represents a fundamental condition for success of ceramic crowns. Since there is potential for optimization, intraoral scanning might enhance preparation quality providing instant visual feedback.

## Introduction

Design and quality of tooth preparation are of fundamental importance for optimum mechanical, biological, and esthetic outcome of dental restorations [1]. The basic principles that must be complied to guarantee success have not considerably altered over time. Among those are suggestions for maximum convergence angle, minimum abutment height, a certain abutment height to base ratio, an adequate resistance form, an appropriate finish line design and location, and a reasonable degree of surface smoothness [2–4].

Preparations for all-ceramic restorations require particular features such as shoulder or chamfer finish lines [5–7] and rounded line angles [2–4]. Specific marginal substance reduction must be complied since different ceramic materials, e.g., feldspathic ceramics, lithium disilicate ceramics, veneered zirconia, and monolithic zirconia, distinctly differ regarding mechanical properties [8].

Modern computer-assisted dentistry not only enables a multitude of novel possibilities but also lays down specific prerequisites. Preparations adequate for digital workflows must largely match the requirements of all-ceramic preparations. Considering the fact that milling accuracy is limited by diameter of cutting instruments, demands related to subtractive fabrication of restorations represent an appropriate depth and evenness of the finish line, and avoidance of sharp edges [4] to waive bur-radius-correction.

Various studies involve the influence of preparations on performance of CAD/CAM restorations [4, 5, 9], the impact of direct digitization (intraoral scanning/IOS) on the quality of CAD/CAM-generated restorations [10], or the effect of CAD/CAM fabrication itself on marginal fit of restorations [11]. Concerning accuracy of intraoral digitization, the methodology has sufficiently proven to be equivalent to conventional techniques [12–14] with the sole exception of full-arch scans [15]. Nevertheless, to the authors’ best knowledge, scarcely any data is available focusing on a “reverse effect” of digitization mode on the quality of preparations for all-ceramic restorations.

This investigation aimed to explore this topic by addressing different parameters critical for the quality of preparations. These determinants include convergence angle, marginal design, marginal reduction, homogeneity of the finish line, and undercuts.

Due to its correlation to retention and resistance of restorations, convergence angle is pivotal in assessment of the quality of preparations and thus well investigated [16, 17]. Recommendations for optimum convergence angles evolved from 2° to 5° to clinically more feasible 6° to 20° [16, 18]. Nonetheless, it has been found that, in dental clinical routine, recommendations for convergence angles are frequently being exceeded [18–20].

Mechanical resistance of all-ceramic crowns depends on preparation design, restorative material used, and appropriate material thickness [5]. Depending on the design of preparation, marginal substance reduction, or finish line depth, is supposed to be between 0.5 and 1.0 mm for all-ceramic crown preparations [2, 21]. Therefore, considerable amounts of tooth substance ranging between 67.5 and 72.3% must be removed [21]. However, a minimum distance of up to 2 mm should be kept between the surface and pulp chamber to protect pulp tissue from unwanted iatrogenic effect [22, 23]. This narrow ridge gives accurate marginal reduction particular importance for the success of the restoration and the integrity of the underlying tooth.

Design and quality of the finish line have proven to show significant impact on the fit of the latter restoration [4]. This subject can be addressed evaluating parameters such as absence or presence of a defined finish line, a reverse bevel, and a uniform and even course of the finish line. Undercuts in preparations must be avoided as their presence necessitates blocking-out which might negatively impact on retention, resistance, and fit of the restoration.

Using fully automated specialized software, this study aimed to compare the influence of intraoral scanning on the quality of all-ceramic single crown preparations regarding different parameters. Preparations indirectly digitized from plaster casts after conventional impression making served as control. Hypothesis was that there will be no difference between the two groups concerning the parameters tested.

## Materials and methods

A total of 690 in vivo preparations were examined. Three hundred sixty-seven data records had been generated digitizing conventional plaster casts (group “indirect digitization”/ID) and 323 data sets had been obtained from performing intraoral scanning (group “intraoral scanning”/IS) (LAVA C.O.S., 3M ESPE, St. Paul, MN, USA). The completely anonymized data records in STL (standard tessellation language) format were provided by a professional milling center (Biodentis, Leipzig, Germany). Data included randomly chosen preparations for all-ceramic restorations from multiple dental practices and practitioners. Ceramic material was not defined in more detail. Each data record contained only one single crown preparation of either a molar or premolar of the maxilla or mandible.

Approval was obtained by the ethics committee of the Ludwig-Maximilians-University Munich.

**Fig. 1:**
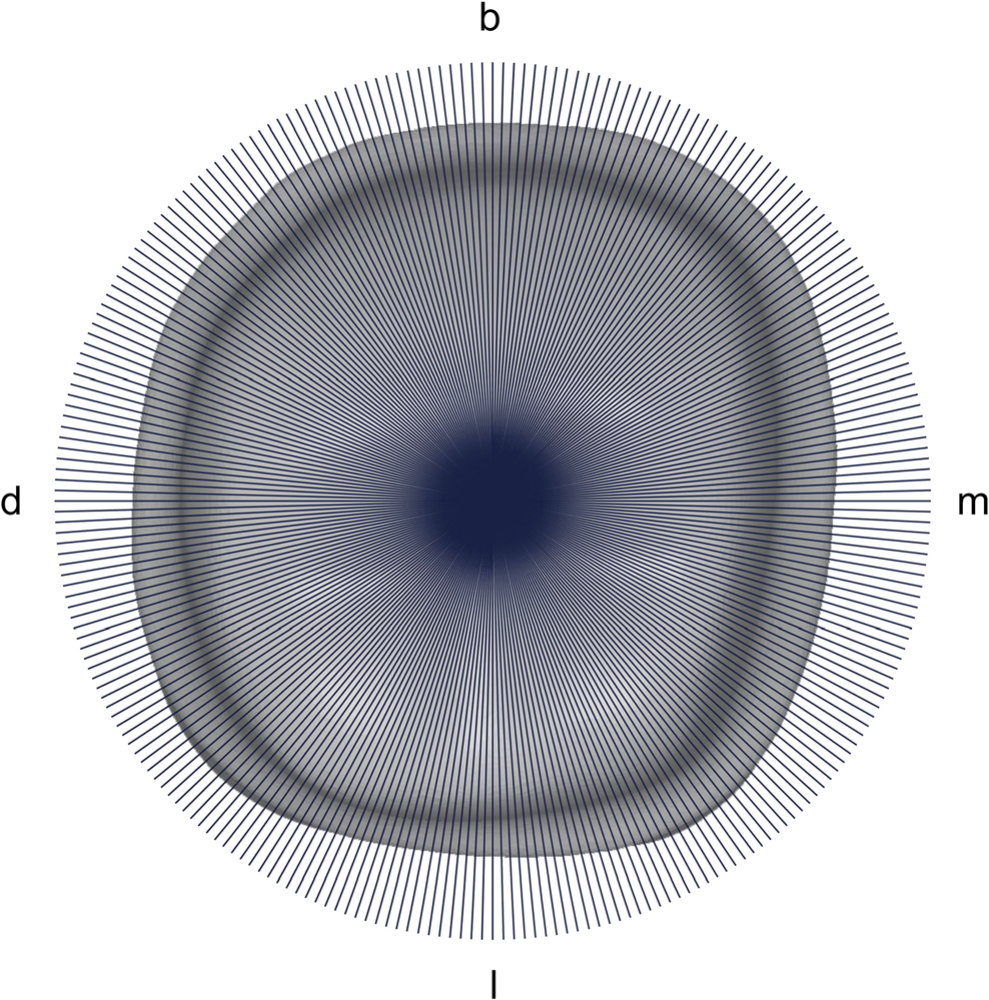
Each specimen was vertically cut along the *z*-axis 360 times resulting in 360 two-dimensional profiles (occlusal aspect)

**Fig. 2:**
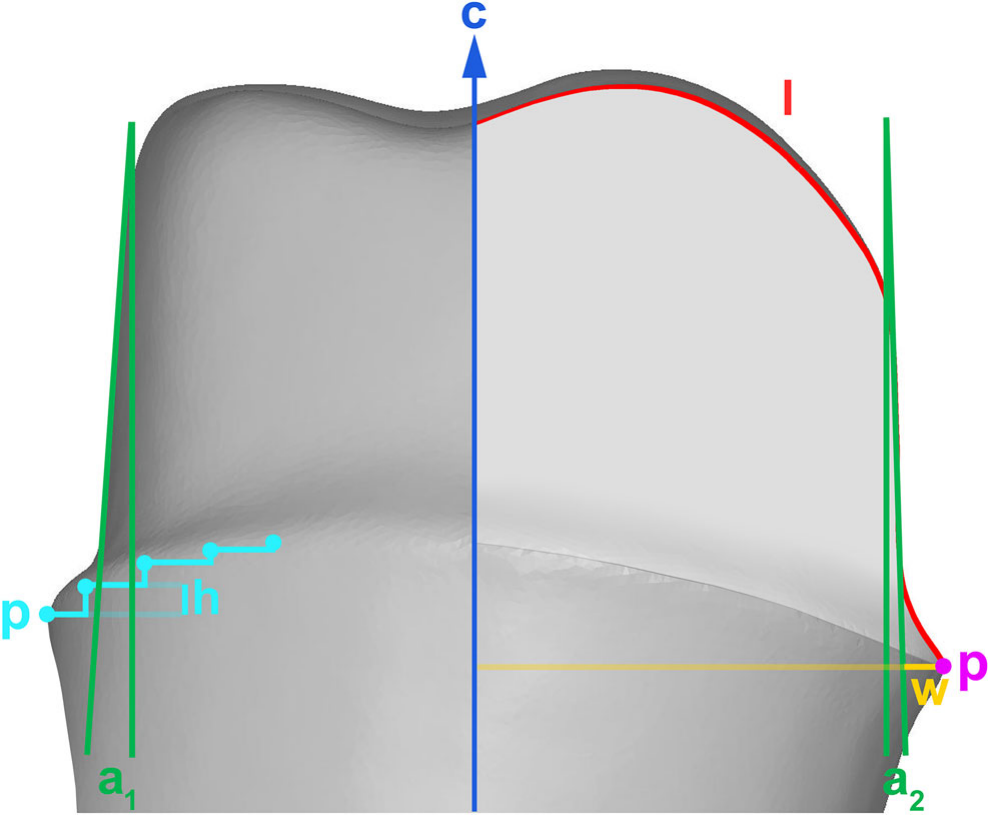
Central axis ((c); blue), profile line ((l); red), preparation angles ((a_1_) and (a_2_); green), marginal reduction ((w); yellow), preparation points ((p); purple/turquoise), and largest difference between two adjacent points ((h); turquoise) (proximal aspect)

All STL data were processed and imported into special inspection software by one skilled professional. The fully automated software (KE.PAS.02) had been developed for this purpose by the Department of Prosthetic Dentistry, University Hospital, LMU Munich. After determination of the central axis, no further operator intervention is necessary. The software sequentially slices every preparation data record 360 times vertically along the central axis producing two-dimensional profiles (Figs. 1 and 2). Predefined parameters are evaluated applying regression analysis and curve sketching to every profile.

### Convergence angle

Automated inspection software generated preparation angles for all 360 profiles per abutment and added tapers of opposing profiles, computing a total of 180 convergence angles per specimen (Fig. 2). The mean convergence angle was calculated for each specimen and subsequently, data were pooled for each group.

Convergence angles were classified “class I” (between 6° and 15°), “class II” (< 6°), or “class III” (> 15°) [18].

### Marginal reduction

Marginal reduction or width of the chamfer was defined as the distance between finish line and preparation tangent along a perpendicular to the central axis (Fig. 2). Three hundred sixty measurements were summarized to calculate the mean for each preparation and then overall values for the groups. Standard deviation was appraised for both groups to determine differences in homogeneity of marginal substance removal.

Marginal reduction was assigned “class I” (between 0.5 and 1 mm), “class II” (> 1 mm), or “class III” (< 0.5 mm).

### Marginal design

Before the marginal design was determined, the existence of a defined finish line was examined (Fig. 2). The parameter “defined finish point” was evaluated for each profile by yes/no decision and resulted in percentage values for each tooth which were subsequently totaled for each group.

When the distance (Fig. 2) between the finish line and preparation tangent along a perpendicular to the central axis was approaching zero, the software recognized the margin to present a “featheredge design.” Yes/no decisions were made for every profile per specimen resulting in percentage values for every specimen and both groups.

**Fig. 3:**
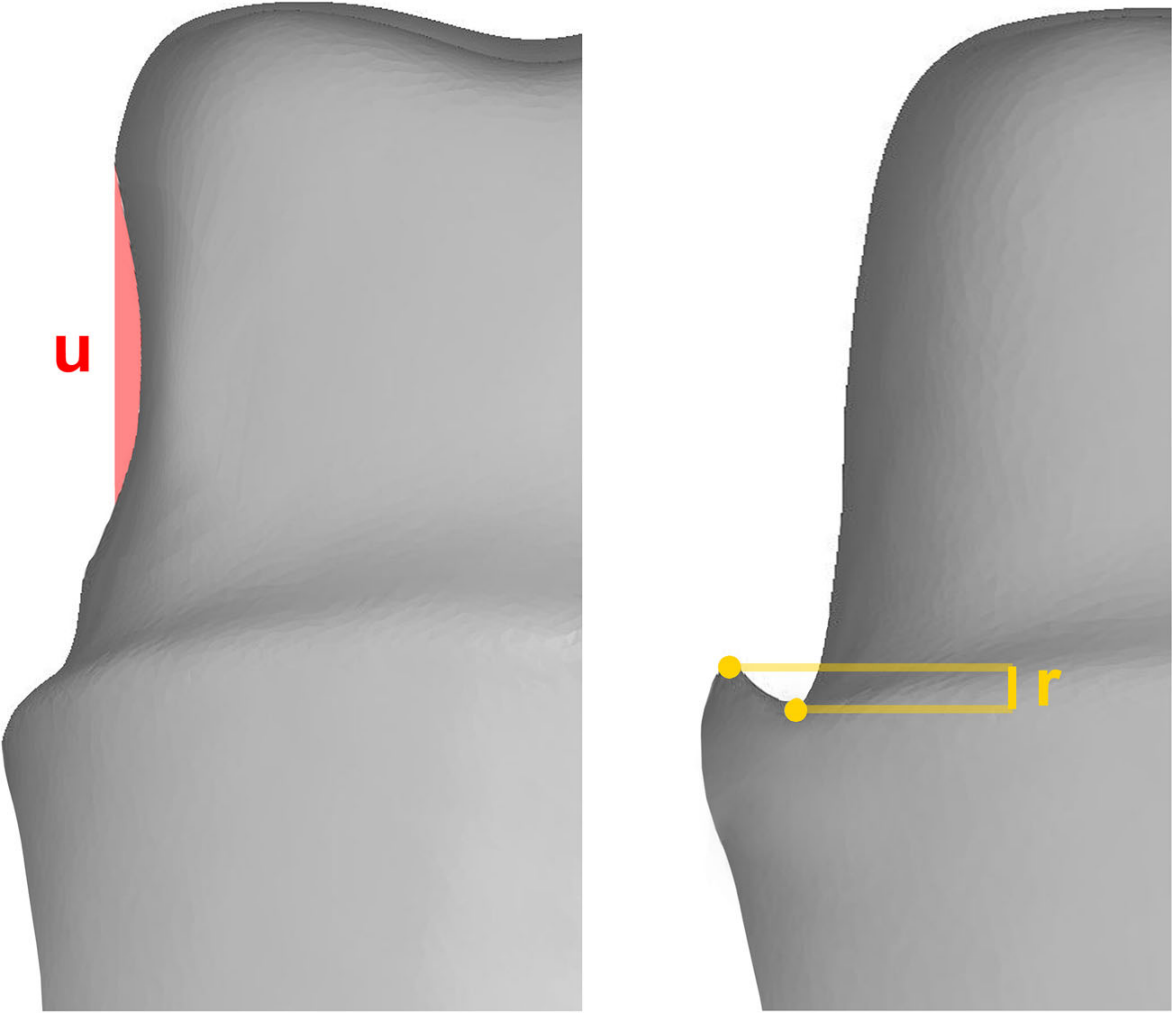
Undercut ((u); red) and a reverse bevel ((r); yellow) (proximal aspects)

“Reverse bevel” was detected by the software when any point along the preparation profile was found to have a lower value on the *z*-axis than the marginal finish point (Fig. 3). The preparation was classified “reverse bevel” as a whole, when one or more of its profiles presented the parameter.

### Homogeneity of the finish line

Assessment of homogeneity of the finish line was based on the largest vertical difference between two adjacent preparation points (Fig. 2), representing the steadiness of the course of the finish line. A value was detected in each specimen and then, overall values were calculated for each group.

### Undercuts

Investigation software identified undercuts as a function of preparation geometry (Fig. 3). Yes/no decisions were made for all 360 profiles per tooth resulting in percentage values for each group.

### Statistical analysis

Kolmogorov-Smirnov test was applied to evaluate, whether the values were distributed normally within the test groups. To compare the groups with each other concerning the different parameters, Mann-Whitney *U* test and Fisher’s exact test were used.

For statistical evaluation, the software SPSS (Statistics 23.0, SPSS Inc., Stanford, USA) was applied and level of significance was set at *p* < 0.05.

## Results

Results are given in Table 1. Mann-Whitney *U* test (MWU) revealed significant difference regarding convergence angle (*p* < 0.001), since a median of 30.79° was found for group ID and of 33.32° for group IS. 8.1% of preparations in group ID and 7.7% in group IS were classified “class I” and therefore optimum, while all others were classified too small (class II) or too large (class III) (Fisher’s exact test, *p* < 0.001).

**Table 1:** Descriptive statistics for convergence angle, marginal reduction, marginal design (defined finish point, featheredge design, reverse bevel), homogeneity of the finish line (maximum difference between two adjacent preparation points), and undercuts

Group	Parameter				
	Convergence angle [%]		Classification of convergence angle [%]		
	Median (IQR)	Mean (SD)	Class I, 6°–15°	Class II, < 6°	Class, III > 15°
ID	30.79 (9.19)	30.48 (6.84)	8.1	3.5	88.4
IS	33.32 (8.98)	32.85 (7.07)	7.7	2.4	89.9
	Marginal reduction [mm]		Classification of marginal reduction [%]		
	Median (IQR)	Mean (SD)	Class I, 0.5–1 mm	Class II, > 1 mm	Class III, < 0.5 mm
ID	0.72 (0.16)	0.71 (0.14)	29.5	26.4	44.1
IS	0.67 (0.19)	0.67 (0.12)	33.1	31.4	35.5
	Marginal design				
	Defined finish point [%]		Featheredge design [%]		Reverse bevel [%]
	Median (IQR)	Mean (SD)	Median (IQR)	Mean (SD)	
ID	96.9 (6.9)	94,4 (6.6)	15.6 (22.2)	20.0 (17.1)	42.3
IS	99.2 (2.8)	97.4 (4.2)	10.0 (18.1)	14.1 (14.4)	35.9
	Homogeneity of the finish line [mm]		Undercuts [%]		
	Median (IQR)	Mean (SD)	Median (IQR)	Mean (IQR)	
ID	0.07 (0.02)	0.07 (0.02)	4.7 (12.5)	8.2 (9.8)	
IS	0.06 (0.01)	0.07 (0.01)	1.4 (9.2)	6.1 (9.4)	

Marginal substance reduction was 0.72 mm in group ID, and thus larger than in group IS (0.67 mm). Statistical significance was found (MWU, *p* < 0.001). Marginal reduction was ideal (class I) in 29.5% in group ID and in 33.1% in group IS. Other preparations showed, with a different distribution of frequency, too much (class II) or too little (class III) substance reduction (Fisher’s exact test, *p* < 0.001). Average standard deviation of marginal reduction was 0.2 for group ID and 0.17 for group IS, displaying higher uniformity in amount of substance removal (MWU, *p* = 0.386).

The parameter “defined finish point” was applied to 96.9% of indirectly digitized (ID) and to 99.2% of intraorally scanned (IS) preparations (MWU, *p* < 0.001). “Featheredge design” was found in 15.6% and 10% of preparation of group ID and group IS, respectively (MWU, *p* < 0.001). “Reverse bevel” was detected in 42.3% of the preparations of group ID and 35.9% of the preparations of group IS (Fisher’s exact test, *p* = 0.211).

 Largest difference between two adjacent points along the finish line was 0.07 mm for group ID and 0.06 mm for group IS (MWU, *p* < 0.001).

Undercuts were found in 4.7% of preparations in group ID and 1.4% of preparations in group IS. Difference was significant (MWU, *p* < 0.001).

## Discussion

Computer-based technologies offer chances enhancing quality management regarding not only standardized production of restorations but also optimization of clinical procedures [24, 25]. Thus, the intention of the current investigation was to evaluate the influence of intraoral scanning on the quality of preparations for all-ceramic single crowns compared with preparations digitized after conventional impression making and plaster casting. Examined parameters included convergence angle, marginal substance reduction, marginal design, and undercuts. Indirectly digitized preparations showed significantly smaller convergence angles, while intraoral scanning demonstrated superior results, although not always significantly, concerning all other tested parameters. Therefore, the hypothesis had to be rejected.

The data examined in this study involved preparations of a premolar or molar randomly taken from anonymized clinical situations provided by multiple dental practices. Therefore, experience of the executing dentists is not known but most probably heterogeneous. This might be qualified by the number of data records and by the fact that the data thus represent a realistic cross-section of performance in daily dental practice. The set of software parameters chosen to evaluate the quality of preparations during this study does not purport to be exhaustive but represents substantial and reasonable factors. Other features such as abutment height, abutment height to base ratio, resistance form, and, although difficulty to achieve, finish line location might also be tested using suitable software.

Assessing the results, it must be noted that differing values in investigations dealing with convergence angles might also be caused by method, for instance, by approach of calculation of the convergence angle [16]. In the present study, overall convergence angle representing an average of convergence angles of all opposite profiles per specimen was calculated. This approach might be considered a limitation since angles of opposing functional surfaces, meaning buccal vs. oral and mesial vs. distal, have not been assessed separately. The protocol, however, provides comprehensive information and seemed reasonable since only posterior teeth were examined.

Convergence angle is of fundamental relevance in evaluating preparation quality. Güth et al. postulated 6–15° to be a reasonable range for convergence angles in all-ceramic single crown preparations, but found average angles of 26.74°, being larger than recommended in 86.7% of investigated preparations under clinical conditions. The authors concluded that dental clinicians seem to face difficulties in meeting recommendations for all-ceramic preparations [18]. The values found in the present investigation, displaying 30.79° in group ID and 33.32° in group IS, with only 8.1% (ID) and 7.7% (IS) being rated “class I” and therefore optimum, are in line with these findings and display considerable potential for improvement. This assessment even applies when compared with less strict recommendations of between 10° and 22° as more recently recommended [2, 19, 26]. Yet, these results are similar to other preceding investigations. Al-Omari et al. found average convergence angles of 32.2° and 34.8° in maxillary and mandibular molars in preparations made by dental students [27]. In a trial of Annerstedt et al., students performed significantly better than practitioners in preparing molars (22.2° vs 26.6°) [28]. This difference is plausible since students work in a learning environment less exposed to time and economic pressure. Winkelmeyer et al. examined STL datasets of, inter alia, zirconia frameworks for single crowns and found median convergence angles of 14.8° for premolars and 24.3° for molars [19]. The reasons preparation angles or convergence angles tend to be larger than requested interrelate with multiple factors. Among those are restricted access and visibility, especially in the molar regions [2, 27], the presence of saliva and blood, and lack of patients’ compliance. Preparations in molars present poorer results in general [29, 30]. Other reasons might be existing defects, malposition of teeth, or tilted tooth angles which frequently occur in vivo. Another reason for the large values in the present study might be the fact that preparations for undefined all-ceramic materials were examined. Preparing teeth for glass-ceramic crowns and trusting on reliability and resilience of adhesive luting might encourage clinicians to be more negligent. The results, not only of the present study, but in general, might imply to prefer adhesive luting over conventional cementation whenever possible from a clinical perspective. A cause for comparably larger values in group IS might be excess compensation by clinicians worrying to not be able to capture all relevant areas with the intraoral scanner. However, the concern is unfounded, as this disadvantage applies for convergence angles of 5° and less [31]. Also, many modern intraoral scanning devices operate according to confocal microscopy rather than triangulation technology, largely dispensing with this drawback.

Optimum marginal substance reduction, or finish line depth, was defined to be between 0.5 and 1.0 mm. Goodacre et al. found that marginal reduction of more than 1.0 mm is unlikely to be achieved in a clinical setting, no matter what the recommendations are [2]. Mean marginal substance reductions in this study of 0.72 mm in group ID and 0.67 mm in group IS confirm those findings. Reduction was graded optimum in 29.5% measurements in group ID and 33.1% in group IS. Average standard deviation of marginal reduction was smaller in group IS showing a more homogenous pattern of substance removal. This uniformity of finish line depth is of importance since supposedly favorable average values do not necessarily take into account areas of major deviation, which might negatively affect the clinical performance of restorations and integrity of underlying teeth. The results for finish line depth are in line with values of preceding studies. Al-Omari et al. found marginal substance reduction in maxillary and mandibular premolars and molars to be between 0.71 and 0.75 mm [27]. Other authors showed finish line depth to range between 0.71 and 0.83 mm [30] and 0.8 and 1.0 mm [32] in premolars and molars depending on measuring site.

A “defined finish point,” tantamount with a visible finish line, was found more often in group IS than in group ID. It is therefore consistent that a larger percentage of “featheredge design” was also found in group ID. Presence of visible finish lines and absence of featheredge design must be perceived positively. Featheredge preparations are not considerably advantageous regarding substance loss [7] and can even be detrimental in terms of biological [33], mechanical, and technical aspects [4]. This is why featheredge design should not be performed on a regular basis in all-ceramic restorations [7]. The parameter “reverse bevel,” which was detected significantly more often in group ID, indicates instable brittle margins and correlates with poorer fit [4].

Homogeneity of the course of the finish line was determined by the largest difference between two adjacent points along the finish line. Group IS displayed the better outcome. A steady and even course of the finish line correlates with the marginal fit of the CAD/CAM-fabricated restoration, which is adversely affected by spiked, beveled, and undulating finish lines that cannot be reproduced by CAD/CAM systems due to limited diameter of cutting instruments [4].

More undercuts were found in group ID, which might suggest an advantage of intraoral scanning, since undercuts hold the risk of reduced quality of fit and retention. Nonetheless, the lower incidence of undercuts might also result from the larger convergence angles in group IS or from the fact that some areas cannot be captured by intraoral scanners and must thus be interpolated. This phenomenon possibly will eliminate some undercuts in datasets disguising their presence and thus distorting the outcome.

The results of this investigation imply that intraoral scanning helps enhance the quality of preparations, even with no automated feedback technology integrated into the scanning device. A reason for this might be the instantaneous visualization of the preparation provided by intraoral scanners. Seeing the preparation in large scale on a computer screen allows for better self-assessment and immediate refinement.

Instant feedback can be assumed to hold remarkable benefits, not only due to the possibility to optimize the quality of preparations. Moreover, the effort of immediately optimizing a preparation and rescanning the relevant areas is considerably less time-consuming than redoing a conventional impression and fabricating a cast or performing an indirect laboratory scan [34]. Receiving concise information on the quality and quantity of preparation errors might result in a steep learning curve which otherwise is unlikely to be expected. This makes such technology equally interesting for pre- and post-graduate dental education as well. Available systems for digital evaluation of preparations have proven to be more reliable than skilled professionals in objectively grading the quality of preparations using specified parameters [20]. This led to have caused positive reactions among users [35]. One major advantage of a software as presented is the dispensability of data superposition with a master reference preparation or tooth as often used in dental education [35, 36]. Hey et al. presented a similar idea in 2013, applying special software that assesses preparation angle, finish line depth, and homogeneity of the finish line, also dispensing with the need for a reference preparation. This is of fundamental importance since objective parameters of universal applicability allow in vivo implementation in the first place. However, operator intervention is also needed for preparation analysis in this approach [37]. The software applied in the present investigation allows for high processing quality and efficiency using a completely automated digital approach. It analyzes as much as 360 profiles per preparation, thus waiving with rough approximation. This ensures most comprehensive quality assessment, guaranteeing low susceptibility to undetected errors. Since evaluation parameters are adjustable, intended restoration design and restorative materials might be factored in. This could help diminish the prevalence of specific preparation errors, create learning experience, and improve the long-term prosthetic outcome.

## Conclusion

Within the limitations of this study, the findings suggest that digitization of preparations using intraoral scanning has a positive effect on the quality of preparations for all-ceramic single crowns in terms of marginal substance reduction, marginal design, homogeneity of finish lines, and frequency of undercuts. It can be deduced that intraoral scanning helps increase preparation quality by visual feedback immediately after scanning. Future effort should focus on developing software tools that automatically analyze preparation design during scanning process and give instant restoration-specific feedback.

## References

[1] Goodacre CJ (2004). Designing tooth preparations for optimal success. Dent Clin North Am.

[2] Goodacre CJ, Campagni WV, Aquilino SA (2001). Tooth preparations for complete crowns: an art form based on scientific principles. J Prosthet Dent.

[3] Podhorsky A, Rehmann P, Wostmann B (2015). Tooth preparation for full-coverage restorations-a literature review. Clin Oral Investig.

[4] Renne W, McGill ST, Forshee KV, DeFee MR, Mennito AS (2012). Predicting marginal fit of CAD/CAM crowns based on the presence or absence of common preparation errors. J Prosthet Dent.

[5] Beuer F, Aggstaller H, Edelhoff D, Gernet W (2008). Effect of preparation design on the fracture resistance of zirconia crown copings. Dent Mater J.

[6] Yu H, Chen YH, Cheng H, Sawase T (2019). Finish-line designs for ceramic crowns: a systematic review and meta-analysis. J Prosthet Dent.

[7] Baltzer A (2008). All-ceramic single-tooth restorations: choosing the material to match the preparation--preparing the tooth to match the material. Int J Comput Dent.

[8] Schwindling FS, Waldecker M, Rammelsberg P, Rues S, Bomicke W (2019). Tooth substance removal for ceramic single crown materials-an in vitro comparison. Clin Oral Investig.

[9] Beuer F, Edelhoff D, Gernet W, Naumann M (2008). Effect of preparation angles on the precision of zirconia crown copings fabricated by CAD/CAM system. Dent Mater J.

[10] Nagarkar SR, Perdigao J, Seong WJ, Theis-Mahon N (2018) Digital versus conventional impressions for full-coverage restorations: a systematic review and meta-analysis. Journal of the American Dental Association (1939) 149 (2):139-147. e131. doi:10.1016/j.adaj.2017.10.001 10.1016/j.adaj.2017.10.001 29389337

[11] Papadiochou S, Pissiotis AL (2018). Marginal adaptation and CAD-CAM technology: a systematic review of restorative material and fabrication techniques. J Prosthet Dent.

[12] Guth JF, Keul C, Stimmelmayr M, Beuer F, Edelhoff D (2013). Accuracy of digital models obtained by direct and indirect data capturing. Clin Oral Investig.

[13] Keul C, Stawarczyk B, Erdelt KJ, Beuer F, Edelhoff D, Guth JF (2014). Fit of 4-unit FDPs made of zirconia and CoCr-alloy after chairside and labside digitalization--a laboratory study. Dent Mater.

[14] Syrek A, Reich G, Ranftl D, Klein C, Cerny B, Brodesser J (2010). Clinical evaluation of all-ceramic crowns fabricated from intraoral digital impressions based on the principle of active wavefront sampling. Journal of dentistry.

[15] Ahlholm P, Sipila K, Vallittu P, Jakonen M, Kotiranta U (2018). Digital versus conventional impressions in fixed prosthodontics: a review. J Prosthodont.

[16] Tiu J, Al-Amleh B, Waddell JN, Duncan WJ (2015). Clinical tooth preparations and associated measuring methods: a systematic review. J Prosthet Dent.

[17] Jorgensen KD (1955). The relationship between retention and convergence angle in cemented veneer crowns. Acta Odontol Scand.

[18] Guth JF, Wallbach J, Stimmelmayr M, Gernet W, Beuer F, Edelhoff D (2013). Computer-aided evaluation of preparations for CAD/CAM-fabricated all-ceramic crowns. Clin Oral Investig.

[19] Winkelmeyer C, Wolfart S, Marotti J (2016). Analysis of tooth preparations for zirconia-based crowns and fixed dental prostheses using stereolithography data sets. J Prosthet Dent.

[20] Kunkel TC, Engelmeier RL, Shah NH (2018). A comparison of crown preparation grading via PrepCheck versus grading by dental school instructors. Int J Comput Dent.

[21] Edelhoff D, Sorensen JA (2002). Tooth structure removal associated with various preparation designs for posterior teeth. Int J Periodontics Restorative Dent.

[22] Evans CD, Wilson PR (1999). The effects of tooth preparation on pressure measured in the pulp chamber: a laboratory study. Int J Prosthodont.

[23] Davis GR, Tayeb RA, Seymour KG, Cherukara GP (2012). Quantification of residual dentine thickness following crown preparation. Journal of dentistry.

[24] Beuer F, Schweiger J, Edelhoff D (2008). Digital dentistry: an overview of recent developments for CAD/CAM generated restorations. Br Dent J.

[25] van Noort R (2012). The future of dental devices is digital. Dent Mater.

[26] Shillingburg HT, Hobo S, Whitsett LD, Jacobi R, Brackett SE (1997) Fundamentals of fixed prosthodontics, vol 3rd ed. Quintessence Publishing, Chicago

[27] Al-Omari WM, Al-Wahadni AM (2004). Convergence angle, occlusal reduction, and finish line depth of full-crown preparations made by dental students. Quintessence Int.

[28] Annerstedt A, Engstrom U, Hansson A, Jansson T, Karlsson S, Liljhagen H, Lindquist E, Rydhammar E, Tyreman-Bandhede M, Svensson P, Wandel U (1996). Axial wall convergence of full veneer crown preparations. Documented for dental students and general practitioners. Acta Odontol Scand.

[29] Nordlander J, Weir D, Stoffer W, Ochi S (1988). The taper of clinical preparations for fixed prosthodontics. J Prosthet Dent.

[30] Poon BK, Smales RJ (2001). Assessment of clinical preparations for single gold and ceramometal crowns. Quintessence Int.

[31] Chan DC, Chung AK, Haines J, Yau EH, Kuo CC (2011). The accuracy of optical scanning: influence of convergence and die preparation. Oper Dent.

[32] Begazo CC, van der Zel JM, van Waas MA, Feilzer AJ (2004). Effectiveness of preparation guidelines for an all-ceramic restorative system. American journal of dentistry.

[33] Lang NP (1995). Periodontal considerations in prosthetic dentistry. Periodontology.

[34] Patzelt SB, Lamprinos C, Stampf S, Att W (2014) The time efficiency of intraoral scanners: an in vitro comparative study. Journal of the American Dental Association (1939) 145 (6):542-551. doi:10.14219/jada.2014.23 10.14219/jada.2014.23 24878708

[35] Hamil LM, Mennito AS, Renne WG, Vuthiganon J (2014). Dental students’ opinions of preparation assessment with E4D compare software versus traditional methods. J Dent Educ.

[36] Arnetzl G, Dornhofer R (2004). PREPassistant: a system for evaluating tooth preparations. Int J Comput Dent.

[37] Hey J, Kupfer P, Urbannek M, Beuer F (2013). Objective analysis of preparations in dental training: development of analytical software. Int J Comput Dent.

